# Repeat Associated Non-AUG Translation (RAN Translation) Dependent on Sequence Downstream of the *ATXN2* CAG Repeat

**DOI:** 10.1371/journal.pone.0128769

**Published:** 2015-06-18

**Authors:** Daniel R. Scoles, Mi H. T. Ho, Warunee Dansithong, Lance T. Pflieger, Lance W. Petersen, Khanh K. Thai, Stefan M. Pulst

**Affiliations:** Department of Neurology, University of Utah, 175 North Medical Drive East, 5th Floor, Salt Lake City, Utah, 84132, United States of America; University of Florida, UNITED STATES

## Abstract

Spinocerebellar ataxia type 2 (SCA2) is a progressive autosomal dominant disorder caused by the expansion of a CAG tract in the *ATXN2* gene. The SCA2 disease phenotype is characterized by cerebellar atrophy, gait ataxia, and slow saccades. *ATXN2* mutation causes gains of toxic and normal functions of the *ATXN2* gene product, ataxin-2, and abnormally slow Purkinje cell firing frequency. Previously we investigated features of *ATXN2* controlling expression and noted expression differences for *ATXN2* constructs with varying CAG lengths, suggestive of repeat associated non-AUG translation (RAN translation). To determine whether RAN translation occurs for *ATXN2* we assembled various *ATXN2* constructs with *ATXN2* tagged by luciferase, HA or FLAG tags, driven by the *CMV* promoter or the *ATXN2* promoter. Luciferase expression from *ATXN2*-luciferase constructs lacking the *ATXN2* start codon was weak vs AUG translation, regardless of promoter type, and did not increase with longer CAG repeat lengths. RAN translation was detected on western blots by the anti-polyglutamine antibody 1C2 for constructs driven by the *CMV* promoter but not the *ATXN2* promoter, and was weaker than AUG translation. Strong RAN translation was also observed when driving the *ATXN2* sequence with the *CMV* promoter with *ATXN2* sequence downstream of the CAG repeat truncated to 18 bp in the polyglutamine frame but not in the polyserine or polyalanine frames. Our data demonstrate that *ATXN2* RAN translation is weak compared to AUG translation and is dependent on *ATXN2* sequences flanking the CAG repeat.

## Introduction

Spinocerebellar ataxia type 2 (SCA2) is an autosomal dominant cerebellar ataxia characterized by progressive degeneration of the cerebellum and parts of the brain stem. SCA2 is caused by CAG repeat expansion in the *ATXN2* gene resulting in polyglutamine (polyQ) expansion in the ataxin-2 protein. The most common normal *ATXN2* allele contains 22 CAGs and repeats of 33 CAGs or greater are pathogenic [1]. Patients with SCA2 are characterized by ataxia slowly progressing with age and slow saccadic eye movements [2], and SCA2 families are characterized by anticipation, whereby disease severity and age of onset correlate with CAG repeat length, which tends to increase generationally [3].

We previously characterized mechanisms of *ATXN2* expression control to identify factors that may be exploited to reduce *ATXN2* expression therapeutically [4]. The study was conducted with the hypothesis that lowering *ATXN2* expression might be therapeutic because of a gene dose-phenotype relationship in polyQ diseases: SCA2 patients and mice homozygous for the mutated *ATXN2* allele have more severe SCA2 phenotypes vs. heterozygous individuals [5,6], and phenotypes of other polyQ disease models are reversible [7–10]. We evaluated numerous *ATXN2*-luciferase (*luc*) constructs with unidirectional and interstitial deletions in the *ATXN2* upstream region and determined that an ETS transcription factor binding site is required for *ATXN2* expression. Our study also investigated the effect of CAG length on *ATXN2* expression. One striking finding was that *ATXN2-luc* with only one CAG was low-expressing compared to any *ATXN2-luc* construct with longer CAGs. Therefore, we investigated this further because of a previous demonstration that expanded CAG repeats in the *ATXN8* gene can initiate protein translation, by so-called repeat associated non-AUG (RAN) translation [11].

For repeat expansion genes, RAN translation is affected by repeat length whereby longer repeats are more susceptible to initiating translation, with no requirement for an AUG start codon [12–22]. RAN translation in all three reading frames (CAG, AGC, and GCA) was observed for CAG expanded *ATXN8*, by constructs lacking a start codon [11]. RAN translation is also initiated by hexanucleotide GGGGCC repeat expansion in intron 1 of the C9FTD/ALS gene *C9ORF72* [19,21,22], and by CGG repeat expansion in the 5’ UTR of *FMR1*, causing fragile X-associated tremor ataxia syndrome (FXTAS) [20]. RAN translation products may form high molecular weight aggregates that can be useful prognosticators of disease and very likely contribute to disease pathology. Accumulations of polyalanine and polyglutamine proteins were observed in disease tissues of SCA8 and DM1 patients [11], accumulations of poly-(glycine-alanine) and poly-(glycine-proline) peptides were observed in multiple CNS tissues from C9FTD/ALS patients [19,21,22], and polyglycine accumulations were observed in FXTAS patient brains [20]. Translation can also occur in the absence of an AUG start codon but not involving repeat expansion [23–29].

Understanding of *ATXN2* RAN translation is important for developing therapeutics that reduce expanded CAG repeat-associated toxic gain of function associated with SCA2. In the present study we evaluated multiple *ATXN2* constructs with varying CAG repeat lengths, with different *ATXN2* sequences downstream of the CAG repeat, and different tags, for the ability to support RAN translation. We demonstrated that the structure of the *ATXN2* sequence downstream of the CAG repeat significantly contributed to the ability for the RNA to undergo RAN translation. Constructs harboring the HA tag were more permissive to RAN translation than those harboring a luciferase tag, and additional *ATXN2* sequence downstream of the CAG repeat abrogated RAN translation. We were not able to demonstrate significant RAN translation by the alternate polyalanine and polyserine frames of the *ATXN2* CAG repeat.

## Materials and Methods

### Ethics Statement

No animal or human participants were used in this research.

### Cloning of *ATXN2-luc* plasmids with start codon substitutions

Plasmid pGL2-5A3 includes a total of 1704 bp of *ATXN2* upstream (1062 bp) and 5’-UTR (642 bp) sequence ahead of the *ATXN2* start codon. Progressing downstream, the construct included *ATXN2* exon 1 encoded sequence through the first CAG of the CAG repeat, followed by the luciferase gene, followed by *ATXN2* downstream sequence including the complete 3’-UTR. pGL2-5B3, pGL2-5C3, and pGL2-5D3 are identical to pGL2-5A3 but include CAG lengths of 22, 57, and 101, respectively and include 108 bp downstream of the CAG repeat. Plasmids pGL2-5A3, pGL2-5B3, pGL2-5C3, and pGL2-5D3 were described previously [4]. Each of these plasmids were altered to include a ATG➔CTG (Met➔Leu) substitution in the *ATXN2* start codon. The resultant constructs are referred to as ATG- or CTG- CAG1, CAG22, CAG57, and CAG101 or CAG102 (sequencing proved that the CTG-CAG102 construct gained 1 CAG relative to its ATG counterpart). The substitutions were made by amplifying the repeat region with forward primer S2-A (5’- TGTATGGGCCCCTCACCCTGTCGCTGAA-3’) that includes an *Apa* I site for cloning and also the ATG➔CTG substitution, and reverse primer S2-B (5’-ccagctcgagggccgaggacgaggagac-3’) that includes a *Xho* I site for cloning. The amplicon was excised from the non-mutant target plasmid with *Apa* I and *Xho* I and the mutant amplicon insert was ligated in place. All constructs were sequenced to verify the presence of the start codon ATG➔CTG substitution and the CAG length. Luciferase assays utilizing these plasmids were controlled with a promoterless luciferase plasmid lacking all *ATXN2* upstream and exon 1 sequence but retaining the *ATXN2* 3’-UTR and downstream sequence after the luciferase gene. The control plasmid was created by excising the *ATXN2* upstream and exon 1 sequence of pGL2-5A3 [4] with *Hpa* I and *Xho* I, filling the *Xho* I sticky end with T4 polymerase digestion, and ligating.

### Cloning of *CMV-ATXN2-luc* plasmids

Plasmid pcDNA3.1-*luc* was first created by amplifying the *luc* insert with primer LucA (5’-GGCCCTCGAGCTGGAAGACG-3’) and LucB (5’-TCGGGGGCCCTTACAATTTGGACTTTCCGCCC-3’), cutting the insert with *Xho* I and *Apa* I, and ligating into vector pcDNA3.1/Hygro(+) prepared with *Xho* I and *Apa* I digestion. *CMV*-*ATXN2-luc* plasmids possess 20 bp of the *ATXN2* sequence immediately upstream of the CAG repeat, through 18 bp downstream of the repeat, and are modeled after plasmids used to study RAN translation in the *HTT*, *JPH3* (*HDL2)*, *MJD1* and *DM1* genes in Zu et al. [11]. To prepare p*CMV*-ATG-*ATXN2-luc* the ATXN2 insert was amplified using primer Bam-A1 (5’-GTAGGGATCCTCACCATGTCGCTGAAGCCC-3’) and primer Xho-B (5’-CTGGCTCGAGGGCAGCCGCGGGCGGCGG-3’), the insert was digested with *Bam* HI and *Xho* I, and ligated into pcDNA3.1-luc prepared with *Bam* HI and *Xho* I digestion. Plasmid p*CMV*-CTG-*ATXN2-luc* was prepared in the same way except using forward primer Bam-A2 (5’- GTAGGGATCCTCACCCTGTCGCTGAAGCCC-3’), which changes the start codon to a CTG. Both p*CMV*-ATG-*ATXN2-luc* and p*CMV*-CTG-*ATXN2-luc* were next modified by inserting a 6xStop cassette between the *CMV* promoter and the *ATXN2* sequence. Two oligos (5’-CTAGCTAGTAGATAGTAGATAGTAGG-3’ and 5’-GATCCCTACTATCTACTATCTACTAG-3’) coding two stop codons in each reading frame were annealed and ligated in p*CMV*-CTG-*ATXN2-luc* prepared with *Nhe* I and *Bam* HI digestion, resulting in plasmid p*CMV*-6xStop-ATG-*ATXN2-luc* and p*CMV*-6xStop-CTG-*ATXN2-luc*. We then created two other plasmids from p*CMV*-6xStop-CTG-*ATXN2-luc*, one with *luc* shifted into the polyserine frame and the other with *luc* shifted into the polyalanine frame. To shift *luc* into the polyserine frame we amplified a fragment of *ATXN2*-*luc* with primer PolyS-For (5’-TGCCCTCGAGACTGGAAGACGC-3’) and primer Luc-Rev (5’-TCGGGGGCCCTTACAATTTGGACTTTCCGCCC-3’) and ligated into p*CMV*-6xStop-CTG-*ATXN2-luc* prepared by *Xho* I and *Apa* I digestion. We named the final construct p*CMV*-6xStop-CTG-*ATXN2(polyS)-luc*. Similarly, to shift *luc* into the polyA frame we amplified a fragment of *ATXN2*-*luc* with primer PolyA-For (5’-TGCCCTCGAGAACTGGAAGACGC-3’) and primer Luc-Rev and ligated into p*CMV*-6xStop-CTG-*ATXN2-luc* prepared by *Xho* I and *Apa* I digestion. We named the construct pCMV-6xStop-CTG-*ATXN2(polyA)-luc*. Sequencing revealed 101 CAG repeats in p*CMV*-6xStop-ATG-*ATXN2-luc*, 102 CAG repeats in p*CMV*-6xStop-ATG-*ATXN2-luc*, 101 AGC repeats in p*CMV*-6xStop-CTG-*ATXN2(polyS)-luc*, and 103 GCA repeats in p*CMV*-6xStop-CTG-*ATXN2(polyA)-luc*.

### Cloning of native promoter *ATXN2-luc* plasmids with luciferase in different frames

Constructs described here allowed for comparing RAN translation in the GCA (polyA), AGC (polyS), and CAG (polyQ) frames of *ATXN2*, upstream of luciferase, driven by the native *ATXN2* promoter. To prepare these constructs we made modifications to pGL2-5D3 (containing 102 CAG repeats in *ATXN2*, previously described [4]) to place *luc* into either the polyA frame or the polyS frame. To prepare an insert for cloning the polyA frame we amplified a fragment of luciferase with forward primer Del1-A (5’-GGCCCTCGAGTGGAAGACGCCAAAAACATA-3’) that includes an *Xho* I site for cloning and a CTG➔TG deletion in the *luc* CTG (the previously altered ATG➔CTG *luc* start codon), and reverse primer Rev-B (5’-CCAGAGGAATTCATTATCAGTGCAATTGTTTT-3’), that primes inside the luciferase gene across a unique *Eco* RI site for cloning. To prepare an insert for cloning the polyS frame we amplified from pGL2-5D3 a fragment of *ATXN2* exon 1 including the CAG repeat using forward primer Ser-A (5’-GGCGTGCGAGCCGGTGTATG-3’) that primes just before the *Apa* I site upstream of the CAG repeat and reverse primer Ser-B (5’-CCTCCTCGAGCGGGCTTGCGGACATTG-3’) that primes downstream of the CAG repeat and includes an *Xho* I site for cloning. This shortens the 108 bp between the end of the CAG repeat and the beginning of the luciferase gene to 34 bp in order to exclude a stop codon in the polyS frame. The insert was ligated between the *Xho* I and *Apa* I sites of pGL2-5D3. Note that the resultant number of repeats in the completed GCA and AGC reporter plasmids is actually one less than the number of CAGs in the initial CAG102-*ATXN2-luc* construct (GCA101 & AGC101). All constructs were sequence verified.

### Cloning *ATXN2*-3T plasmids

To assess RAN translation by western blotting we created *ATXN2* expression plasmids each with three epitope tags (3T tag), with one epitope in each of the three frames. The 3T tag included the HA tag in the polyQ frame, the FLAG tag in the polyS frame, and the MYC tag in the polyA frame. To accomplish this, both p*CMV*-6xStop-ATG-*ATXN2-luc* and p*CMV*-6xStop-CTG-*ATXN2-luc* were modified by replacing *luc* with a 3T tag. Two oligos, 3T-For (5’- TCGAGTACCCATACGATGTTCCAGATTACGCTGGATTACAAGGATGACGACGATAAGAGAACAGAAACTGATCTCTGAAGAAGACCTGTAAGGGCC -3’) and 3T-Rev (5’- CTTACAGGTCTTCTTCAGAGATCAGTTTCTGTTCTCTTATCGTCGTCATCCTTGTAATCCAGCGTAATCTGGAACATCGTATGGGTAC -3’) encoding the 3T cassette were annealed and ligated in p*CMV*-6xStop-ATG-*ATXN2-luc* and p*CMV*-6xStop-CTG-*ATXN2-luc* prepared with *Xho* I and *Apa* I digestion, resulting in plasmids p*CMV*-6xStop-ATG-*ATXN2-3T* and p*CMV*-6xStop-CTG-*ATXN2-3T*, respectively.

### Cloning of HA and FLAG-HA series of *ATXN2* plasmids

Plasmids pATG-*ATXN2-HA*, pATG-*ATXN2-FLAG-HA*, p*CMV-ATG-ATXN2-HA* and p*CMV-ATG-ATXN2-FLAG-HA* with the *ATXN2* ATG start codon and plasmids pCTG-*ATXN2-HA*, pCTG-*ATXN2-FLAG-HA*, p*CMV-CTG-ATXN2-HA* and p*CMV-CTG-ATXN2-FLAG-HA* with the start codon substituted with CTG were constructed in a stepwise manner described here. Both of p*CMV*-6xStop-ATG-*ATXN2-3T* and p*CMV*-6xStop-CTG-*ATXN2-3T* were modified to include a longer sequence of *ATXN2* downstream of the CAG repeat, tagged with a single HA epitope. This was done by amplifying an insert fragment of *ATXN2* with primer ATX-for (5’-TGCCCTCGAGAATGTCCGCAAGCCCG-3’) and primer ATX-HA-rev (5’- AAACGGGCCCTTAAGCGTAATCTGGAACATCGTATGGGTATTTGTACTGGGCACTTGACTC-3’), digesting the insert with *Xho* I and *Apa* I, and ligating the insert into either p*CMV*-6xStop-ATG-*ATXN2-3T* or p*CMV*-6xStop-CTG-*ATXN2-3T* prepared by *Xho* I and *Apa* I digestion, resulting in plasmids p*CMV*-ATG-*ATXN2-HA* and p*CMV*-CTG-*ATXN2-HA*. Sequencing demonstrated these plasmids contained 101 and 102 CAG repeats, respectively, and 801 bp of *ATXN2* sequence downstream of the CAG repeat, exclusive of the *Xho* I site (originating from from pGL2-5A3). Plasmid pATG-*ATXN2*-HA was prepared by excising the *CMV* promoter in p*CMV*-ATG-*ATXN2-HA* by digestion with *Nru* I and *Nhe* I and replacing it with the *ATXN2* promoter obtained by PCR using primer pm1001b (5’-TGCTTCGCGAGGCCCCAGAGGCTGAGAC-3’) and primer pm1002 (TCAGGCTAGCGGTGAGGGGCCCATACAC), with template pGL2-5A3. This *ATXN2* promoter fragment, designated *ATXN2p*, includes 96 bp of *ATXN2* upstream sequence ahead of the 5’-UTR transcription start site (a total of 738 bp upstream of the translation initiation site) and is longer than the minimal length required for *ATXN2* expression and longer lengths can drive *ATXN2* expression more weakly in HEK293 cells (see [4]). Plasmid pCTG-*ATXN2*-HA was prepared in the same way except the modification was to plasmid p*CMV*-CTG-*ATXN2-HA*. Sequencing verified that both plasmids pATG-*ATXN2-HA and* pCTG-*ATXN2-HA* contained 101 and 102 CAG repeats, respectively. Next we modified each of p*CMV*-ATG-*ATXN2-HA*, p*CMV*-CTG-*ATXN2-HA*, pATG-*ATXN2-HA and* pCTG-*ATXN2-HA* by including an in-frame FLAG epitope 18 bp downstream of the CAG repeat. Because modifications made at this position resulted in CAG repeat contraction we did this stepwise. We prepared a double-stranded insert expressing the FLAG epitope by annealing oligo FLAG1A (5’-TCGAGCTCGATTACAAGGATGACGACGATAAGC-3’) and FLAG2B (5’-TCGAGCTTATCGTCGTCATCCTTGTAATCGAGC-3’) and ligating the annealed insert into the *Xho* I site of pCTG-*ATXN2-HA* downstream of the CAG repeat. By screening multiple constructs we obtained one containing 91 CAG repeats, designated pCTG-*ATXN2-FLAG-HA*. We then used pCTG-*ATXN2-FLAG-HA* as a template and amplified an *ATXN2* fragment including the complete CAG repeat, using primers BamATG-For (5’- ATAGTAGGGATCCTCACCATGTCGCTGAAG-3’) and Bam-REV (5’- CGAAACATATCATTGGGATCCCATCCATTA-3’), by ligating the amplicon into p*CMV*-ATG-*ATXN2-HA* prepared by *Bam* HI digestion, resulting in plasmid p*CMV*-ATG-*ATXN2-FLAG-HA*. To prepare p*CMV*-CTG-*ATXN2-FLAG-HA* we cut a CAG repeat containing fragment from plasmid pCTG-*ATXN2-FLAG-HA* with *Bam* HI and ligated it into plasmid p*CMV*-CTG-*ATXN2-FLAG-HA* prepared by *Bam* HI digestion. Finally, pATG-*ATXN2-FLAG-HA* was prepared by obtaining a CAG repeat containing fragment from p*CMV*-ATG-*ATXN2-FLAG-HA* with *Nhe* I and *Aar* I, and ligating it into pCTG-*ATXN2-HA* cut with the same two enzymes. Each of the plasmids pATG-*ATXN2-FLAG-HA*, p*CMV*-ATG-*ATXN2-FLAG-HA*, and p*CMV*-CTG-*ATXN2-FLAG-HA* were verified by sequencing to contain 91 repeats like the ancestor plasmid pCTG-*ATXN2-FLAG-HA*.

### Luciferase assays

HEK293T cells (ATCC) were grown in Dulbecco’s Modified Eagle Medium (DMEM) with 10% fetal bovine serum and 1X penicillin/streptomycin. Cells were transfected using Xfect Transfection Reagent following the vender’s protocol (Clontech). Transfections were conducted in triplicate wells of a 24-well plate. Transfections included 125 ng of luciferase reporter plasmid and 40 ng of pRL-SV40 (Promega). Assays were performed in triplicate per transfection after 24 or 48 h transfection using Dual-Glo Luciferase Assay System (Promega), recording relative light units (RLUs) from firefly luciferase and Renilla luciferase on a multimode plate reader (Beckman DT880). Values were reported as the mean ± standard deviation (SD) of the ratios of firefly luciferase / Renilla Luc (FLuc / RLuc), with n = 3 transfections for the calculation of SD. Each experiment was repeated at least three times.

### Western blot assays

Proteins were separated on precast polyacrylamide gels (Bio-Rad), transferred to Hybond (Amersham) and detected by ECL (Amersham). Antibodies included goat anti-luciferase (Rockland Immunochemicals), mouse 5TF1-1C2 anti-polyglutamine antibody (Millipore), monoclonal mouse anti-**β**-actin-peroxidase (Sigma), monoclonal rabbit anti-c-Myc (Cell Signaling), mouse anti-HA-peroxidase (Roche), mouse anti-FLAG M2 (Stratagene), anti-hygromycin phosphotransferase (My Biosource Inc.). Secondary antibodies included peroxidase conjugated anti-mouse (Vector laboratories), and peroxidase conjugated donkey anti-goat and peroxidase conjugated donkey anti-rabbit (Jackson ImmunoResearch Laboratories).

### Real time PCR

HEK293T cells were cultured and transfected as above, in triplicate. Total RNA was extracted from cultured cells using the RNeasy Mini Kit (Qiagen Inc.) according to the manufacturer’s protocol. DNAse I treated RNAs were used to synthesize cDNA using ProtoScript cDNA synthesis kit (New England Biolabs Inc.). Two sets of primers were used for RT-PCR including luciferase primers GL2luc-2F (5’-ATCCGGAAGCGACCAACGCC-3’) & GL2luc-2R (5’-GTCGGGAAGACCTGCCACGC-3’) and *GAPDH* primers GAPDH-F (5’-GAAGGTGAAGGTCGGAGTCAACG-3’) & GAPDH-R (5’-GAAGATGGTGATGGGATTTCC-3’). Quantitative RT-PCR (qPCR) was performed in Bio-Rad CFX96 (Bio-Rad Inc.) with the Power SYBR Green PCR Master Mix (Applied Biosystems Inc.). PCR reaction mixtures contained SYBR Green PCR Master Mix and 0.5 pmol primers. PCR amplification was carried out for 45 cycles. Cycling parameters were denaturation (95°C for 10 s), annealing (60°C for 10 s), extension (72°C for 40 s). The threshold cycle for each sample was chosen from the linear range and converted to a starting quantity by interpolation from a standard curve run on the same plate for each set of primers. Luciferase expression level was normalized to *GAPDH*.

## Results

### Investigation of RAN translation using *ATXN2* plasmids driven by native promoter

**Fig 1:**
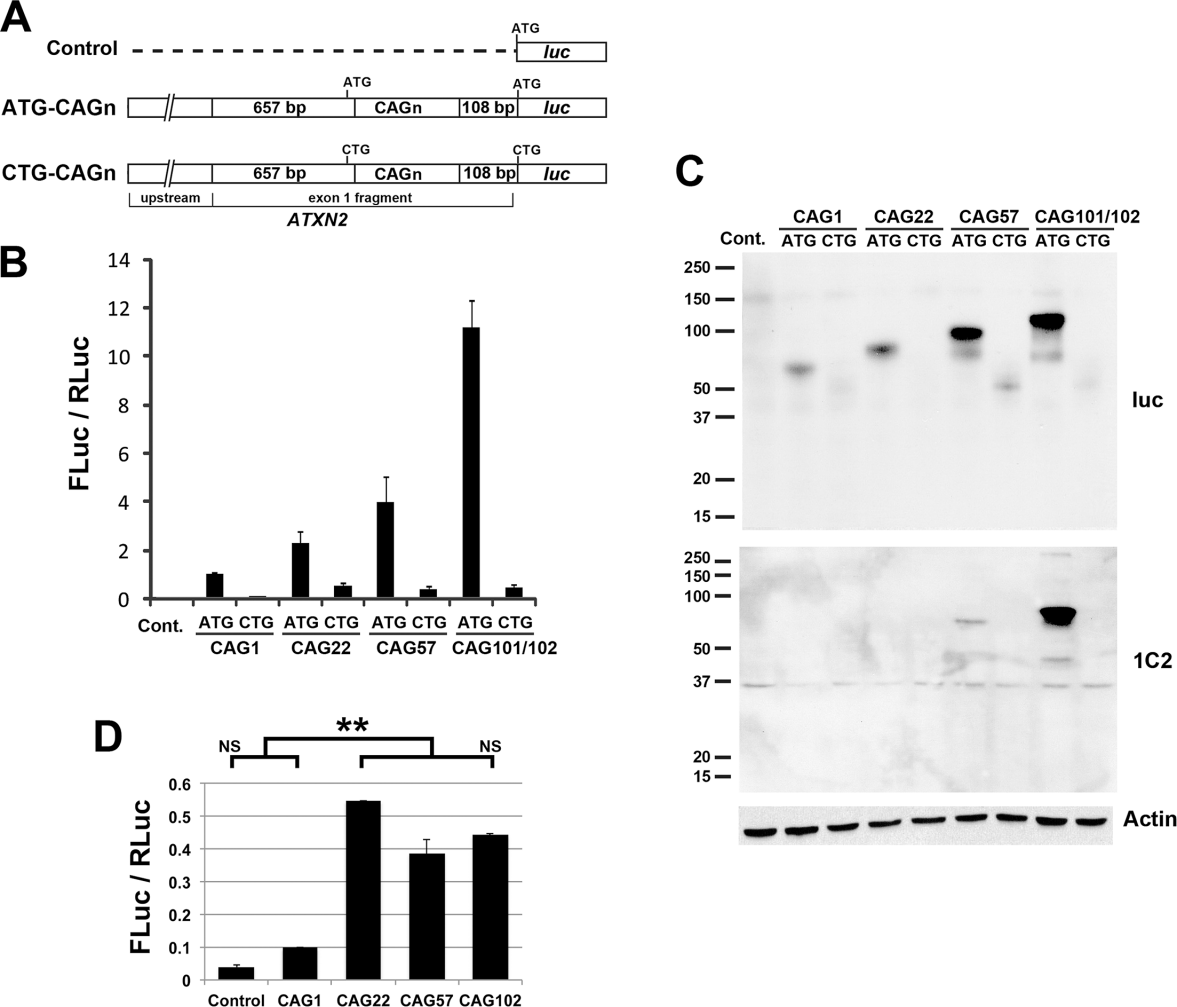
*ATXN2-luc* expression driven by the native *ATXN2* promoter, dependent upon CAG length and the presence of a start codon. (A) Plasmid constructs used in luciferase assays. (B) Luciferase assays to evaluate *ATXN2* expression driven by 1062 bp of its native upstream sequence, demonstrated increasing expression with increasing CAG length (ATG constructs). When the start codon was mutated, expression significantly higher than the control was observed only for *ATXN2*s with CAG repeat lengths of 57 or 102 (CTG constructs). For the longest repeat expression was 25-fold reduced when the start codon was substituted with CTG. Values are mean±SD of three independent experiments. All constructs were cotransfected with SV40-Renilla luciferase and values are represented as mean FLuc / RLuc, the ratio of firefly luciferase to Renilla luciferase. (C) RAN translation products were not observed by western blotting using anti-luciferase (luc) or 1C2 antibodies. Note that polyglutamine proteins detected with the 1C2 anti-polyglutamine antibody are more easily seen as the length of the polyglutamine is increased. Loading was controlled by detecting actin. The mobilities of the smaller ataxin-2-luciferase bands are not consistent with RAN translation bands. (D) Analysis of the luciferase assay results for only the CTG-*ATXN2-luc* constructs in B revealed significantly increased expression for constructs with 22 or greater CAG repeats but no increasing luciferase expression with increasing CAG repeat length. P<0.001 (**), Bonferroni post-hoc probability of significance. Assays utilized HEK293T cells with assays made 24 hrs after transfection.

In order to evaluate the hypothesis that expanded CAG repeats in *ATXN2* might initiate translation, we prepared multiple *ATXN2-luc* plasmids with different CAG length with and without the start codon (Fig 1A). The plasmids used in this experiment included 1062 bp of *ATXN2* upstream sequence to drive expression because our initial intention was to describe RAN translation in the presence of the native *ATXN2* promoter. We performed luciferase assays with these plasmids using HEK293T cells (Fig 1B). We observed increase in luciferase expression with increasing CAG length for plasmids with the *ATXN2* ATG start codon. When the start codon was substituted (ATG➔CTG) the expression of *ATXN2-luc* with 1 CAG was only slightly higher than the vector control, but the expression level of *ATXN2-luc* constructs with 22, 57, or 102 CAG repeats were significantly higher, but still remarkably low compared to those constructs with the non-mutant start codon. This increase in expression might be attributed to RAN translation, but we did not see further increase in *ATXN2-luc* expression with increasing CAG repeat length (Fig 1D). We observed no corresponding RAN translation bands by western blotting using anti-luciferase antibody or anti-polyglutamine 1C2 antibody (Fig 1C).

### Investigation of RAN translation using *CMV*-*ATXN2-luc* plasmids

**Fig 2:**
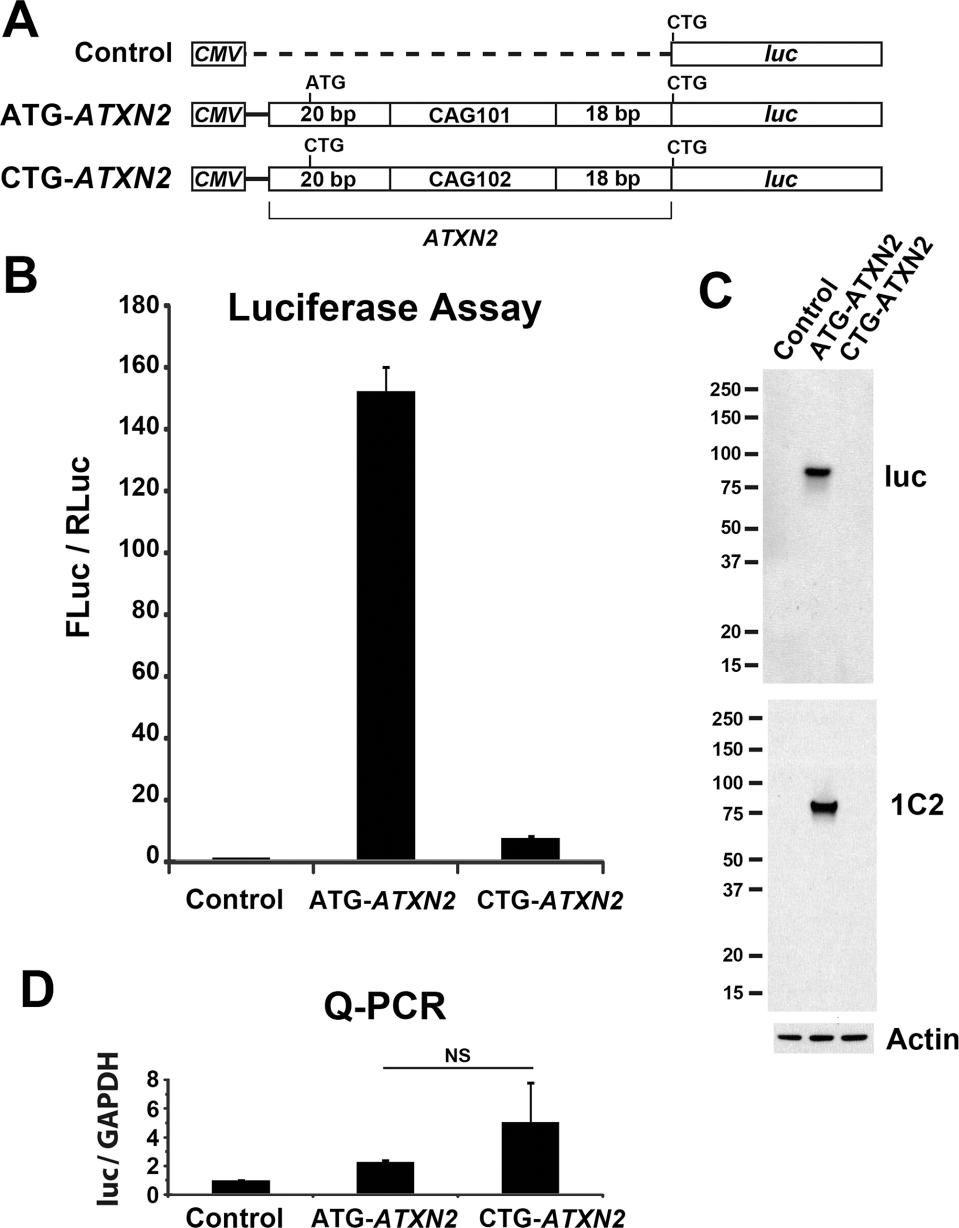
Expression of *ATXN2-luc* driven by the *CMV* promoter. (A) Plasmid constructs used in luciferase assays. (B) Strong luciferase expression was observed for *CMV*-*ATXN2* with the non-mutant ATG. When the ATG was mutated to CTG expression was 7-fold higher than the vector control but 20-fold lower compared to when the ATG was present, consistent with the presence of weak RAN translation for *CMV-ATXN2-luc*. Values are mean±SD of three independent experiments. (C) RAN translation products were not observed by western blotting using anti-luciferase or anti-1C2 antibodies. Loading was controlled by detecting actin. (D) The reduced expression could not be attributed to reduced transcription, because qPCR assays comparing the expression of the transcripts indicated a non-significant trend toward higher transcription when the ATG➔CTG substitution was present. Assays utilized HEK293T cells with assays made 24 hrs after transfection.

To make *ATXN2* RAN translation more easily observed we increased transcript expression using the *CMV* promoter, as was done by Zu et al. [11]. Zu et al. studied RAN translation for *HTT*, *HDL2*, *MJD1*, and *DM1*, by creating plasmid constructs that included long CAG repeats preceded by 20 bp of gene-specific sequence, followed by antibody epitope tags. We followed this same approach for *ATXN2* by creating *CMV-ATXN2-luc* plasmids with and without the ATG start codon, including only 20 bp *ATXN2* sequence upstream of an expanded CAG (CAG101 or CAG102) repeat (Fig 2A). Luciferase assays using these constructs in HEK293T demonstrated strong *ATXN2-luc* expression in the presence of the ATG start codon that was reduced by 20 fold when the start codon was substituted with CTG (Fig 2B). When the start codon was mutated, *ATXN2-luc* expression remained 7 fold higher than background. This result was essentially the same as observed when using *ATXN2*-luc constructs driven by the native *ATXN2* promoter. Despite use of a *CMV* promoter, putative RAN translation for CAG102 *ATXN2-luc* remained weak, and like for native-promoter-*ATXN2*-luc we were unable to detect the RAN translation protein products by western blotting, using anti-luciferase or anti-1C2 antibodies (Fig 1C). We also conducted quantitative PCR (qPCR) using RNAs from transfected HEK293T cells, to evaluate luciferase transcript abundance relative to GAPDH, demonstrating no evidence for the changes observed by luciferase assays or western blotting that could be accounted for by altered transcription (Fig 2D). One notable observation was that the fold-difference for CAG100 *ATXN2-luc* with vs. without a start codon driven by the native promoter was the same as the fold-difference for CAG101/102 *ATXN2-luc* with vs. without a start codon driven by the *CMV* promoter, at 20–25 fold reduction when the start codon was deleted (Fig 1B and Fig 2B). This indicated that there was little advantage of adding a *CMV* promoter to evaluating luciferase expression from these constructs.

### Investigation of RAN translation using HA and FLAG HA epitope tagged *ATXN2* plasmids

**Fig 3:**
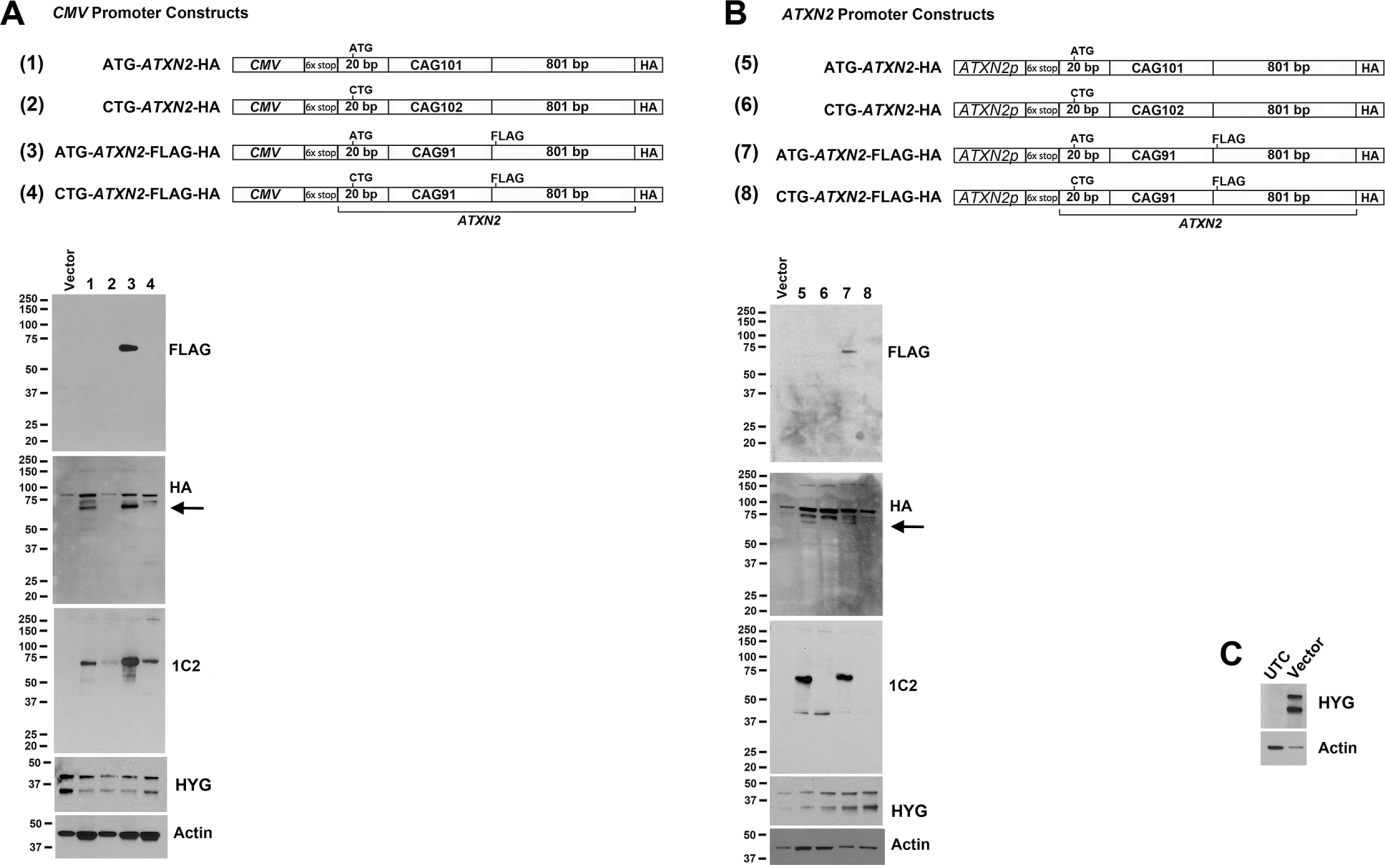
*ATXN2* RAN translation was observed for *ATXN2* sequences with 91 or 102 CAG repeats with C-terminal epitopes driven by the *CMV* promoter but not the native *ATXN2* promoter. (A) *CMV* promoter driven *ATXN2* constructs including the *ATXN2* ATG start codon expressed proteins as expected, detected on western blots by anti-HA (lanes 1 and 3) and anti-FLAG when the epitope was included (lane 3), and anti-1C2 antibodies. When the *ATXN2* start codon was changed to CTG, *ATXN2* RAN translation bands were detected by anti-1C2 (lanes 2 and 4), but RAN translation products were not by anti-FLAG and for anti-HA the faintest RAN translation band is present for construct #4 but not #2. (B) *ATXN2* promoter (*ATXN2p*) driven *ATXN2* constructs including the *ATXN2* ATG start codon expressed proteins as expected, detected on western blots by anti-HA (lanes 5 and 7) and anti-FLAG when the epitope was included (lane 7), and anti-1C2 antibodies. When the *ATXN2* start codon was changed to CTG, *ATXN2* RAN translation bands were not observable by anti-1C2, anti-HA, or anti-FLAG antibodies (lanes 6 and 8). All constructs include the hygromycin phosphotransferase (HYG) gene, and uniformity of plasmid transfection and loading was ensured in A and B by detecting blots with anti-HYG and anti-Actin. Note that the intensity of upper bands detected by the anti-HA antibody follow actin band intensity but not HYG indicating that these are non-specific bands. Arrows indicate the specific bands detected by the anti-HA antibody. (C) Anti-HYG detected a doublet of bands in lysates from transfected cells that was absent in untransfected cells (UTC). For each of A, B, and C, we utilized HEK293T cells and 48 hr transfections.

To further evaluate *ATXN2* RAN translation we replaced the luciferase tag in our constructs with epitope tagged *ATXN2* sequence. Following Zu et al. [11], we also inserted a 6X stop codon cassette (two stops in each frame) upstream of the *ATXN2* start codon. Downstream of the CAG repeat included 801 bp of *ATXN2* sequence with 101/102 CAG repeats tagged with a single C-terminal HA tag or an additional in-frame FLAG epitope 18 bp downstream of the *ATXN2* sequence with 91 CAG repeats. Construct maps are provided in Fig 3A and 3B. We tested expression of these constructs in HEK293T cells by western blotting using anti-FLAG, anti-HA, and anti-1C2 antibodies with the *ATXN2* start codon present or substituted with CTG, when driven by the *CMV* promoter or an *ATXN2* promoter fragment (738 bp of upstream and 5’-UTR sequence). When constructs were driven by the *CMV* promoter expression was observed for all when the ATG start codon was present, including anti-HA, anti-1C2, and also for anti-FLAG when the FLAG epitope was present (Fig 3A). When the *ATXN2* start codon was substituted by CTG we observed RAN translation using the 1C2 antibody (Fig 3A). The detected RAN translation bands were notably weaker than expression when the ATG start codon was present, and the RAN translation bands could not be visualized by western blotting using anti-FLAG, and when anti-HA was used we observed no RAN translation band for construct #2 in Fig 3A but there was an exceptionally weak band for construct #4. These results demonstrated that RAN translation is not favored vs AUG translation. Nearly identical results were obtained when the *ATXN2* promoter was utilized to drive the expression of the otherwise identical constructs, except that no RAN translation bands could be observed when the ATG start codon was substituted to CTG (Fig 3B). Transfection and western blot loading was controlled by the use of anti-hygromycin phosphotransferase (HYG) (all plasmids contained the hygromycin resistance gene) and anti-Actin antibodies (Fig 3A–3C). Note that for each construct in Fig 3 we evaluated ≥ 5 independent plasmid preparations by western blotting and all were fully sequenced, to guard against CAG repeat mosaicism.

### *ATXN2* RAN translation initiated in alternate *ATXN2* reading frames

**Fig 4:**
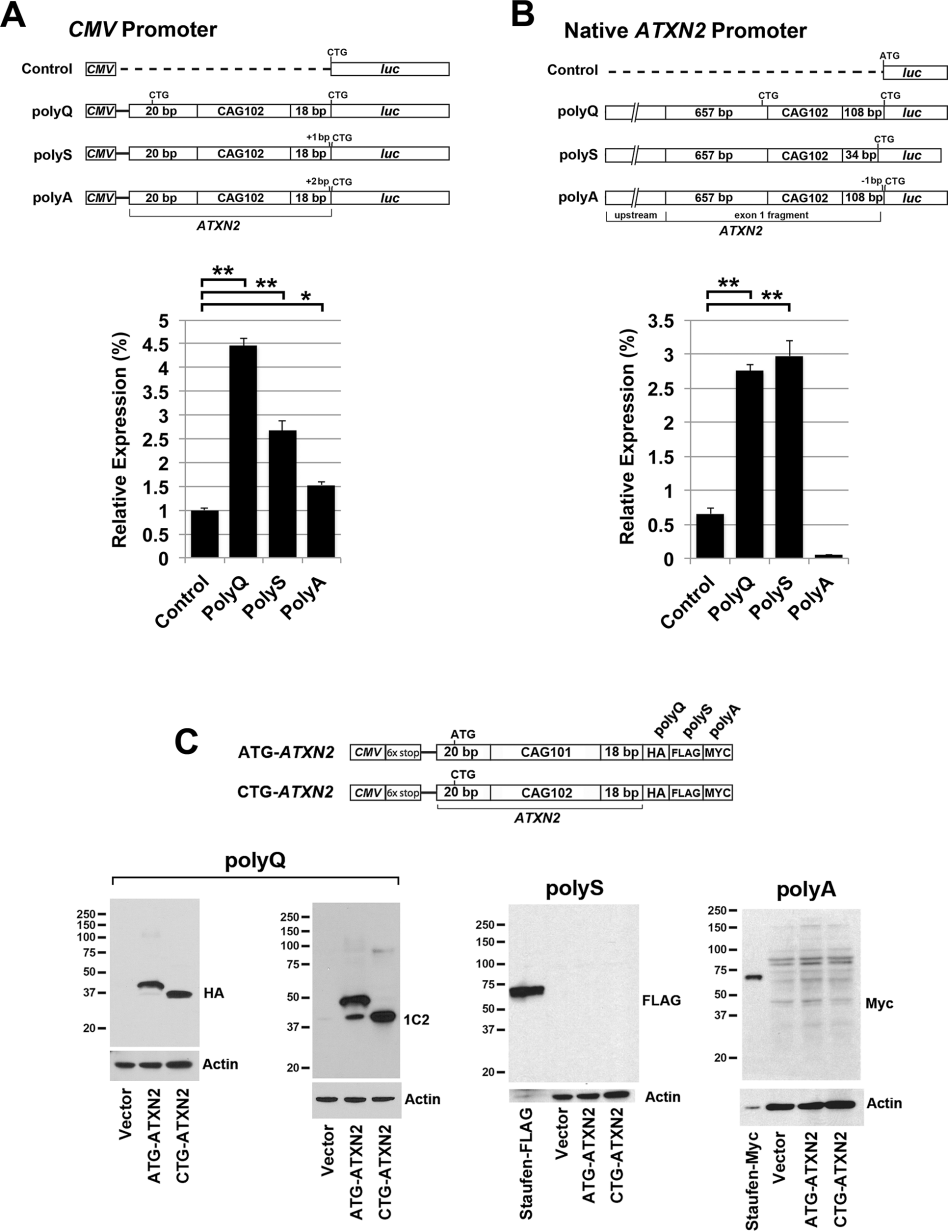
RAN translation in *ATXN2* alternate reading frames. (A and B) Luciferase assays performed using *ATXN2* constructs with expression driven by (A) the *CMV* promoter or (B) the native *ATXN2* promoter, with luciferase shifted into the PolyQ, PolyS, and PolyA frame. In all cases no *ATXN2* ATG start codons were included upstream of the CAG repeat that were in frame with luciferase, and the luciferase start codon was changed to CTG. Expression is shown as a percentage of the value determined for the *CMV* driven (A) or *ATXN2* promoter driven (B) polyglutamine frame constructs with the inclusion of the *ATXN2* ATG start codon. Bonferroni post-hoc probabilities of significance were P<0.001 (**) and P<0.01 (*). Values shown are mean±SD. The experiment was replicated 3 times. Assays were performed in HEK293T cells. Western blots of protein lysates from B and C revealed no bands detectable with anti-luciferase antibody. (C) *ATXN2* constructs with a 18 bp fragment of *ATXN2* sequence downstream of the CAG repeat followed by the 3T tag and western blotting detection. When the ATG was changed to CTG, a band resulting from *ATXN2* RAN translation was readily detected by western blotting of HEK293T cell lysates with anti-HA and anti-1C2 antibodies. No RAN translation bands were observed in the polyS or polyA frames. Note that only background banding was observed when using the anti-Myc antibody. Staufen-FLAG and Staufen-Myc was included as a positive control for FLAG and Myc detections.

We evaluated RAN translation in HEK293T cells in the alternative *ATXN2* reading frames by luciferase assays. We used constructs with the *ATXN2* start codon substituted to CTG and a C-terminal luciferase gene in the polyglutamine (polyQ) frame or shifted into the polyserine (polyS) or polyalanine (polyA) frame, with expression driven by the *CMV* promoter or the *ATXN2* promoter. When constructs were driven by the *CMV* promoter expression in either of the polyQ, polyS, or polyA frames was significantly higher than the vector control but remained low, at 4.5, 2.5 and 1.5%, respectively, of the expression observed in the polyQ frame when the ATG start codon was present (Fig 4A). When constructs were driven by the *ATXN2* promoter expression greater than the control was only observed when luciferase was in the polyQ and polyS frames, and expression was only 3% of that observed in the polyQ frame when the ATG start codon was present (Fig 4B). Western blotting using HEK293T cell lysates and anti-luciferase antibodies revealed no bands for these constructs. All constructs that produced no expression were subjected to additional bidirectional full-length sequence verification.

We also investigated *ATXN2* RAN translation in alternate reading frames by a western blot strategy using a *CMV* promoter to drive up expression of C-terminally tagged short *ATXN2* proteins. The constructs that we used included the 6x stop cassette separating the *CMV* promoter and the *ATXN2* start codon, and had 101 (or 102) CAG repeats followed by 18 bp of *ATXN2* sequence, and a C-terminal 3T tag. The 3T tag was similar to that used in Zu et al. [11], except that the epitope order was HA (in frame with polyQ), FLAG (in frame with polyS), and MYC (in frame with polyA). Using the HA antibody reporting expression in the polyQ frame, we observed strong expression from *CMV*-*ATXN2*-3T plasmids, regardless of whether the start codon was substituted with CTG or not (Fig 4C). When the start codon was substituted to CTG a single RAN translation band was observed by expression in the polyQ frame only, at approximately 37 kDa. When the start codon was not substituted, however, we observed a predominant 40 kDa band, and the 37 kDa band was very weak. This indicated that translation initiation was highly favored from the AUG start codon vs RAN translation initiating from the CAG repeat. Western blotting of proteins expressed from *CMV*-*ATXN2*-3T plasmids using anti-FLAG reporting translation in the polyS frame, and anti-Myc reporting translation in the polyA frame revealed no bands above background, consistent with absence of detectable RAN translation in the polyS or polyA frames (Fig 4C).

## Discussion

This study was initiated to determine whether RAN translation could be demonstrated for the *ATXN2* gene. The motivation for this study was our observation that an *ATXN2-luc* construct with a single CAG repeat produced 50–75% less luciferase expression of an otherwise identical *ATXN2*-*luc* construct with 22 CAG repeats, the most common human wildtype allele (Fig 1 and [4]), without mRNA abundance explaining the difference [4]. We observed only weak evidence for RAN translation for *ATXN2-luc* constructs lacking the ATG start codon. We observed that *ATXN2* sequences driven by the strong *CMV* promoter could undergo RAN translation, but RAN translation was inhibited when the *ATXN2* sequence downstream of the CAG repeat was lengthened. The study demonstrated that RAN translation for *ATXN2* was determined by the sequence flanking the CAG repeat and was not favored compared to AUG translation.

### Evidence for *ATXN2* RAN translation

Our study demonstrated a potential for the *ATXN2* gene to undergo RAN translation. However, the conditions that permit RAN translation are only partly revealed by the results of this study. In seeking evidence for RAN translation for *ATXN2*, we undertook a number of strategies: increase of the CAG repeat length in constructs lacking the start codon, use of *CMV* and endogenous *ATXN2* promoters, alterations of the length of sequence downstream of the CAG repeat, and the use of luciferase vs small epitope tags.

We observed lack of increasing expression with increasing CAG repeat length for *ATXN2-luc* constructs with the start codon mutated, not supporting RAN translation from *ATXN2-luc* constructs. When we increased the length of the CAG repeat in *ATXN2-luc* constructs we observed progressively increased *ATXN2-luc* expression. Previously we observed no transcriptional differences among constructs with different CAG lengths and concluded that translational regulation might account for the observed expression differences [4]. The implication was that the expanded *ATXN2* CAG repeat might enhance CAP mediated translation initiation or progression, or that the repeat might support RAN translation. But for the same constructs without the ATG start codon, we were unable to observe RAN translation bands by western blotting, and there was no progressively increasing *ATXN2-luc* expression (Fig 1). Typically, RAN translation strength increases with the length of the CAG repeat, resulting in the production of multiple homopolymeric proteins [12]. The lack of increasing expression with CAG repeat length for ATG mutated *ATXN2-luc* constructs argues against RAN translation produced by these constructs, yet elimination of the start codon did not entirely abolish expression but reduced it to 5 times the background seen with transfections performed using the vector control. We then attempted to observe RAN translation by driving up the *ATXN2-luc* expression for the construct with 102 CAG repeats and by replacing the native *ATXN2* promoter with the *CMV* promoter. The use of the *CMV* promoter resulted in an order of magnitude increase in *ATXN2-luc* expression, but once again when the ATG was eliminated the expression was reduced to about 5 times background. There was no evidence for RAN translation bands by western blotting (Fig 2).

*ATXN2* RAN translation was observed when we used the *CMV* promoter to drive *ATXN2* sequences tagged with C-terminal epitopes. *CMV* constructs of *ATXN2* including -20 bp upstream of the CAG repeat thru 801 bp downstream of the CAG repeat with 101/102 CAG repeats expressed *ATXN2* proteins. We tagged these constructs with the HA epitope and we also include an in-frame FLAG tag for some constructs with 91 CAG repeats in effort to not overlook RAN translation bands that might form incomplete proteins, although none such bands were observed. When the start codon was eliminated we observed ATXN2 bands that were detectable with the anti-polyglutamine antibody 1C2, but the expression of these RAN translation bands were considerably weaker than when driven by the ATG start codon and not detected using the anti-FLAG antibody, and only the faintest RAN-translation band was detected using anti-HA antibody (Fig 3A). We also prepared an identical series of epitope-tagged *ATXN2* constructs driven by the *ATXN2* promoter (738 bp of upstream and 5’-UTR sequence). When the *ATXN2* promoter was utilized we were unable to observe evidence of RAN translation (Fig 3B). The inability to observe RAN translation when using the *ATXN2* promoter was likely due to weaker expression (the strength of the *CMV* promoter vs the *ATXN2* promoter is indicated by comparing Figs 1B and 2B). The overall result demonstrates that *ATXN2* AUG translation is strongly favored over *ATXN2* RAN translation.

### RAN translation in alternate reading frames

We evaluated RAN translation in the alternate reading frames in two ways, by luciferase assays using *ATXN2-luc* constructs in which *luc* had been shifted into the alternate reading frames, and western blotting using epitope tags. While luciferase expression from the CTG-*ATXN2-luc* constructs was low (Figs 1 & 2), this nevertheless provided an opportunity to investigate RAN translation driven by the *ATXN2* promoter in alternate reading frames. When luciferase was shifted into either the polyQ, polyS, or polyA frame in CTG-*ATXN2-luc* constructs driven by the *CMV* promoter, expression for each of these constructs was significantly higher than for the vector control background (Fig 4A). CTG-*ATXN2-luc* expression was also significantly higher when luciferase was shifted into the polyQ and polyS frames but not the polyA frame when driven by the *ATXN2* promoter (Fig 4B). However, apparent RAN translation from each of these constructs was weak compared to AUG translation (under 5% of AUG translation in the polyQ frame), and the relative differences of translation among the reading frames was not the same as that observed for constructs including a truncated *ATXN2* sequence downstream of the CAG repeat, followed by a C-terminally positioned 3T epitope tag in place of luciferase. The 3T tag includes three epitopes positioned into each of the three reading frames, that has been used previously for the study of RAN translation [11]. For CMV-*ATXN2*-3T with 102 CAG repeats, western blot detection revealed strong RAN translation in the polyQ frame (Fig 4C). Expression from the *CMV*-*ATXN2*-3T construct in the polyQ frame was as strong as the complementary AUG translation. Additionally, a small quantity of the RAN translation product was observed even when the start codon was retained, indicating that the retention of the start codon does not prevent downstream RAN translation for the *CMV*-*ATXN2*-3T construct. This was not predicted based on a discussion of Kozak consensus sequence impact on translation initiation site codon usage indicating that it is unlikely that a preinitiation complex would bypass a strong upstream initiation codon in order to utilize a suboptimal one downstream [29]. The observation of RAN translation for the *CMV-ATXN2*-3T construct in the polyQ frame demonstrated that truncation of the *ATXN2* sequence downstream of the CAG repeat is more permissive of RAN translation, or conversely that the *ATXN2* sequence downstream of the CAG repeat inhibits *ATXN2* RAN translation. We concluded that the minimal luciferase expression that we observed from CTG-*ATXN2-luc* constructs was not due to RAN translation because increasing CAG repeat lengths did not result in increasing RAN translation (Fig 1D), and expression was generally the same among the three frames (Fig 4A and 4B) unlike expression differences observed among the three frames when using the *CMV-ATXN2*-3T construct.

### Ribosomal frameshifting in *ATXN2*

Translation artifacts observed by Toulouse et al. (2005) from expanded *MJD-1* CAG repeats were attributed to ribosomal frameshifting [30]. The expanded CAG repeat of the *MJD-1* gene, which causes SCA3, produced polyalanine and polyserine proteins in a repeat length-dependent manner. Consistent with RAN translation, polyalanine protein expression increased with longer repeat lengths, but polyserine protein expression was low. For *MJD-1* the requirement for a start codon was not determined [30], therefore it remains unclear if the translation of *MJD-1* in the alternative reading frames were initiated by RAN translation. However, treatment with anisomycin and sparsomycin, drugs that affect programmed ribosomal frameshifting, modulated expression of polyalanine and polyserine proteins expressed by *MJD-1* [30]. Our study produced no evidence for frameshifting contributing to the translation of the expanded CAG repeat in *ATXN2*, because the *ATXN2-luc* constructs with luciferase in the polyS and polyA frames retained the native *ATXN2* start codon in the polyQ frame, but only low luciferase levels of expression were observed for those constructs (Fig 4B), and for the ATG-*ATXN2*-3T construct expression was only observed in the polyQ frame (Fig 4C).

### Sequence factors influencing RAN translation

Initiation of RAN translation is influenced by secondary structure of sequences flanking the CAG repeat. The formation of secondary RNA structures appears to be critical, because hairpin-forming CAG repeats undergo RAN translation, while non-hairpin-forming CAA repeats of a similar length do not [11]. Hairpin structures are also important for CAG associated frameshifting, as hairpin-forming CAG repeats undergo frameshifting, constrasted with non-hairpin-forming CAA repeats of a similar length [30]. Additionally, the GGGGCC hexanucleotide repeat of *C9ORF72* forms hairpin structures that are even more stable than those formed from CAG repeats. GGGGCC hairpin structures become even more stable as repeat length increases [21]. G-quadruplex structures also contribute to RAN translation, are repeat length- and flanking sequence-dependent, and the hexanucleotide repeat of *C9ORF72* and the CGG repeat of *FMR1* both form G-quadruplex structures [20,31]. Constructs lacking an ATG initiation codon, containing an expanded CAG repeat with 20 bp of upstream sequences from the *HTT*, *HDL2*, *MJD1*, *DM1* genes, respectively, all produced RAN translation proteins, but with variable efficiency [11]. This is consistent with evidence that the flanking sequence of repeating units influences the threshold stability of hairpin structures [32]. In our study, the ability to observe *ATXN2* RAN translation was promoted by close proximity of the short 3T epitope tag to the CAG repeat, but inhibited by inclusion of 801 bp of *ATXN2* downstream of the CAG repeat. We conclude that inclusion of the 801 bp of *ATXN2* downstream of the CAG repeat induced secondary RNA structures that inhibited RAN translation. The *ATXN2* mRNA might undergo RAN translation *in vivo* that is regulated by RNA secondary structural shifts mediated by microRNAs or RNA binding protein interactions [33].

### RAN Translation and Pathogenesis

RAN translation protein products are now known to contribute to pathogenesis in multiple diseases. *ATXN8* RAN translation products accumulate in Purkinje cells of SCA8 patient brains [11]. *C9ORF72* RAN translation products accumulate in ALS/FTD patient brains [19,22]. Expanded CGG repeat RAN translation products were identified in *FXTAS* patient brains [20]. For *ATXN2*, we demonstrated that RAN translation is possible when the gene is truncated. This raises the possibility that *ATXN2* RAN translation might occur under the right circumstances, *in vivo*.
